# 5-aminoisoquinoline improves renal function and fibrosis during recovery phase of cisplatin-induced acute kidney injury in rats

**DOI:** 10.1042/BSR20171313

**Published:** 2018-04-27

**Authors:** Andrés Quesada, Francisco O’Valle, Sebastián Montoro-Molina, Mercedes Gómez-Morales, Mercedes Caba-Molina, Juan Francisco González, María C. de Gracia, Antonio Osuna, Félix Vargas, Rosemary Wangensteen

**Affiliations:** 1 Área de Fisiología, Departamento de Ciencias de la Salud, Universidad de Jaén, Jaén, Spain; 2 Departamento de Anatomía Patológica, Facultad de Medicina, Universidad de Granada, Granada, Spain; 3 Instituto de Biomedicina y Medicina Regenerativa (IBIMER), Granada, Spain; 4 Unidad de Nefrología, FIBAO, Hospital Virgen de las Nieves, Granada, Spain; 5 Departamento de Fisiología, Facultad de Medicina, Universidad de Granada, Granada, Spain; 6 Instituto Biosanitario de Granada (IBS), Granada, Spain

**Keywords:** 5-aminoisoquinoline, cisplatin, kidney, renal fibrosis, renal dysfunction

## Abstract

The aim of the present study is to analyze the effects of 5-aminoisoquinoline (5-AIQ), a poly(ADP-ribose) polymerase-1 (PARP1) inhibitor, over renal dysfunction and fibrosis during recovery phase of cisplatin (CisPt)-induced acute kidney injury (AKI) in rats. Male Wistar rats were distributed in three groups (*n*=8 each group): control, CisPt, and CisPt + 5-AIQ. Control and CisPt groups received a subcutaneous injection of either saline or 7 mg/kg CisPt, respectively. CisPt + 5-AIQ group received two intraperitoneal injections of 10 mg/kg 5-AIQ 2 h before and 24 h after CisPt treatment. Thirteen days after the treatment, rats were housed in metabolic cages and 24-h urine collection was made. At day 14, CisPt-treated rats showed increased diuresis, N-acetyl-β-d-glucosaminidase (NAG) excretion, glucosuria and sodium fractional excretion (NaFE), and decreased creatinine clearance (CrCl). 5-AIQ significantly increased CrCl and decreased NAG excretion, glucosuria, and NaFE. In plasma, CisPt increased sodium, urea, and creatinine concentrations, while 5-AIQ treatment decreased these variables to the levels of control group. 5-AIQ completely prevented the body weight loss evoked by CisPt treatment. CisPt also induced an increased renal expression of PAR polymer, α-smooth muscle actin (α-SMA), transforming growth factor-β1 (TGF-β1), and collagen-IV. These variables were decreased in CisPt + 5-AIQ group. Tubular lesions and renal fibrosis were also decreased by 5-AIQ treatment. We conclude that inhibition of PARP1 with 5-AIQ can attenuate long-term nephrotoxic effects associated with the CisPt treatment, preventing renal dysfunction and body weight decrease and ameliorating tubular lesions and collagen deposition.

## Introduction

Cisplatin (CisPt) is an antitumoral agent that induces direct proximal tubular nephrotoxicity in both humans and animals [1,2]. This side effect limits the use of CisPt because one-third of the patients develop renal dysfunction after CisPt treatment [3–5]. Although the mechanisms implicated in CisPt nephrotoxicity are not completely elucidated, it has been published that poly(ADP-ribose) polymerase 1 (PARP1) is an important mediator of CisPt-induced renal tubular necrosis and inflammation [6,7].

PARP1 is a nuclear enzyme that repairs DNA strands in homeostatic conditions, converting NAD^+^ into ADP-ribose. Nevertheless, its overactivation in several pathological conditions induces a depletion of NAD^+^ and ATP that triggers cellular death and up-regulation of key inflammatory pathways [8,9].

Inhibitors of PARP1 alone or in combination with DNA-damaging anticancer agents have been proven to facilitate tumor cell death [10–14] and it has been demonstrated a synergistic chemosensitivity with CisPt in breast cancer cell lines [15]. Therefore, inhibition of PARP1 in CisPt treatment can be of great interest since it could potentiate antitumoral effects of CisPt. Moreover, pharmacologic inhibition or genetic deletion of PARP1 also attenuated CisPt-induced increased oxidative and nitrative stress [16] that has been involved in CisPt-induced renal interstitial fibrosis [17].

These previous reports seem to indicate that PARP1 plays a role in CisPt-associated nephrotoxicity. Thus, inhibitors of PARP1 have been demonstrated to improve short-term renal dysfunction in CisPt-treated mice [7,16], but the effects of PARP1 inhibition over long-term renal dysfunction and fibrosis that are associated with CisPt treatment have not been studied. Therefore, the aim of this work is to study if the inhibition of PARP1 with 5-aminoisoquinoline (5-AIQ) prevented long-term CisPt-associated nephrotoxicity, analyzing the effects of this treatment over renal function and histopathological lesions, and focussing our attention on the mechanisms underlying renal fibrosis in this model. To do it, we analyzed renal expression of α-smooth muscle actin (α-SMA) as an index of myofibroblast activation, transforming growth factor-β1 (TGF-β1) as a mediator of interstitial fibrosis [18], and collagen-IV deposition.

## Materials and methods

### Ethical approval

All experimental procedures were performed according to the European Union Guidelines to the Care and Use of Laboratory Animals and approved by the Ethical Committee of the University of Jaén.

### Animals and drugs

Twenty-four male Wistar rats weighing 242 ± 6.69 g were purchased from Harlan Laboratories (Barcelona, Spain). These rats were kept in a room maintained at 24 ± 1°C and humidity of 55 ± 10% with a 12-h light/dark cycle and had a free access to rat chow and tap water. CisPt and 5-AIQ were purchased from Sigma-Aldrich (Darmstadt, Germany).

### Experimental protocols

Rats were distributed in three groups: Control, CisPt, and CisPt + 5-AIQ (*n*=8 each group). Control and CisPt groups received a subcutaneous injection of either saline or 7 mg/kg of CisPt, respectively. CisPt was dissolved in saline at a concentration of 0.35 mg/ml under sonication. CisPt + 5-AIQ group received two i.p. injections of 10 mg/kg of 5-AIQ dissolved in saline 2 h before and 24 h after CisPt treatment. Volumes of injections were calculated according to the weight of each animal. Control and CisPt groups were also injected with the same proportional volume of saline. Thirteen days after CisPt or saline administration, rats were housed in metabolic cages and 24-h urine collection was made. Urine samples were centrifuged for 15 min at 1000 ***g***and frozen at −80°C until assay. Blood samples were obtained from left ventricle under anesthesia (pentobarbital, 50 mg/kg, i.p.), centrifuged for 15 min at 1000 ***g***, and stored at −80°C. Kidneys were removed and weighted. One kidney was fixed in 10% neutral-buffered formaldehyde solution for 48 h and subsequently placed in 70% ethanol for histological studies.

### Analytical procedures

Proteinuria, urine creatinine, and, N-acetyl-β-d-glucosaminidase (NAG) were determined in urine samples by an autoanalyzer Spin120. Plasma creatinine and urea were also measured in this instrument. Reagents for proteinuria (ref. MI1001025), urea (ref. MI41041), and creatinine-Jaffé method (ref. MI1001111) were provided by Spinreact (Barcelona, Spain). Reagent for NAG (ref. DZ062A-K) was purchased from Dyazyme Laboratories (Poway, CA, U.S.A.).

Plasma and urinary sodium were quantified with a sodium selective electrode in the EasyElectrolyte instrument (Medica Corporation, Bedford, MA, U.S.A.).

Renal expression of PAR polymer was measured to evaluate the ability of 5-AIQ to inhibit PARP1 using indirect ELISA. α-SMA, TGF-β1, and collagen-IV were also measured in renal tissue by indirect ELISA. Tissues were homogenized in 50 mM Tris/HCl (pH 7.4) and centrifuged for 15 min at 1000*** g***. Fifty microliters of supernatants containing 10 µg/ml protein were fixed in a 96-well plate (Nunc Maxisorp, VWR International Eurolab, Barcelona, Spain) overnight at 4°C. After blocking with 100 µl PBS containing 5% albumin during 2 h at room temperature, the plate was probed overnight at 4°C with 50 µl of 1 µg/ml of rabbit anti-PAR (Trevigen, Gaithersburg, MD, U.S.A.), anti α-SMA (Proteintech, Manchester, U.K.), anti-collagen-IV (Proteintech, Manchester, U.K.), and anti-TGF-β1 as primary antibodies and 1 h at 37°C with 50 µl of 0.2 µg/ml mouse anti-rabbit horseradish peroxidase-linked IgG antibody (KPL Inc., Gaithersburg, MD, U.S.A.) as a secondary antibody. Plate was washed after blocking and after each antibody incubation with 250 µl of 50 mM Tris (pH 7.4) containing 0.05% Tween-20 during 3 min for five times. After washing, plate was incubated with 50 µl of 3,3′,5,5′-tetramethylbenzidine (TMB) for 30 min at 37°C, and reaction was stopped by adding 50 µl of HCl 1 N. Absorbance was read at a wavelength of 450 nm with a reference wavelength of 590 nm. All samples were analyzed in duplicate. Results were expressed as percentage of the mean absorbance of the control group. Tissue protein was determined with the DC Protein Assay kit (Bio-Rad, Hercules, CA, U.S.A.).

### Histopathological analysis

For conventional morphology, the kidneys were buffered with 10% formaldehyde-fixed during 48 h at room temperature, dehydrated, and paraffin-embedded in an automatic tissue processor (Thermo Scientific Excelsior AS, Thermo Fisher Scientific, Waltham, Massachusetts, U.S.A.) and transversal sections in horizontal plane were deparaffinized in xylol (three passes of 5 min) and re-hydrated in ethanol of decreasing gradations (absolute, 96%, and 70%, two passes of 3 min, respectively). Kidneys’ sections were stained with Hematoxylin and Eosin, Masson’s trichrome, and periodic acid-Schiff stain (PAS). The morphological study was done in blinded fashion on 4-μm sections with BX42 light microscopy (Olympus Optical Company, Ltd., Tokyo, Japan), using the most appropriate stain for each lesion (S3 dysplasia, acute tubular necrosis, apoptosis, casts, tubular atrophy, tubular dilation, vacuolization, inflammatory infiltrate, tubular mitosis). The severity of lesions was calculated semiquantitatively using a 0–3 scale (0, absence; 1, mild (<10% of juxtamedullary proximal tubules, vessels, or glomeruli involved); 2, moderate (10–25%); 3, severe (>25%)).

### Morphometrical analysis

Kidney samples fixed in buffered with 10% formalin were embedded in paraffin and serially sectioned at 5-µm thickness. Afterward, they were stained with 1% Picro Sirius Red F3BA (Gurr, BDH Chemicals Ltd., Poole, U.K.) for image analysis quantitation. To improve staining, tissue sections were kept after deparaffination for 3–5 days in 70% ethanol as mordant. Picro Sirius Red stains connective fibers deep red and cell nuclei and cytoplasmatic structures light red/bright yellow [19]. Twenty images of corticomedullary junction per kidney were acquired using an IF 550 green optical filter with illumination intensity values slightly above those used for normal observation with a digital camera 3CCD (DP70) coupled to an Olympus BX-42 microscope (Olympus Optical Company). Twenty images of corticomedullary junction per kidney were acquired using polarized light. Histologic images of kidney biopsies were converted into black and white at 8-bit intensity resolution (256 gray levels) with a global magnification of 200× and normalized with Adobe Photoshop software (Adobe Systems Software, Ireland). To assess the fibrosis, we made a macro that included a semiautomatic thresholding of the total of images per group of rats simultaneously with ImageJ software (National Institutes of Health, U.S.A.).

### Statistical analysis

StatGraphics software was used for statistical analysis. One-way ANOVA and Bonferroni’s test were used for the analysis of variables with normal distribution. Mann–Whitney W (Wilcoxon) test was used to analyze the differences in tubular lesions. A *P*-value of 0.05 was accepted as statistical significance threshold.

Regression analysis was made with StatGraphics software, selecting a linear model to correlate Sodium Fractional Excretion (NaFE) and creatinine clearance (CrCl) in Control and CisPt + 5-AIQ groups and a multiplicative model in CisPt group.

## Results

### Morphologic and metabolic variables

Treatment with CisPt evoked a progressive loss of body mass along the 2 weeks of experiment, and also increased kidney weight in comparison with control animals. Both morphologic alterations were prevented by treatment with 5-AIQ (Table 1).

**Table 1:** Morphologic and metabolic variables determined 14 days after CisPt injection in experimental groups

	Control	CisPt	CisPt + 5-AIQ
Body weight (g)	272 ± 8.3	240 ± 7.2*	265 ± 6.0
Body weight gain (%)	10.5 ± 0.6	−7.3 ± 0.2^‡^	10.1 ± 0.2^║^
Kidney weight (mg)	958 ± 49	1210 ± 70*	1099 ± 58
KW/BW (mg/g)	3.51 ± 0.09	5.04 ± 0.25^‡^	4.13 ± 0.15^§^
Water intake (ml/100 g/day)	9.34 ± 0.41	14.5 ± 0.71^‡^	19.9 ± 0.65^‡,║^
Diuresis (ml/100 g/day)	3.81 ± 0.56	8.51 ± 0.63^‡^	7.25 ± 0.75^†^
Water balance (ml/100 g/day)	5.53 ± 0.29	5.98 ± 0.28	12.7 ± 0.77^‡,║^

Data are expressed as means ± S.E.M. Abbreviation: KW/BW, kidney weight/body weight *ratio*. *P<0.05. ^†^*P*<0.01. ^‡^*P*<0.001 compared with control group. ^§^*P*<0.01. ^║^*P*<0.001 compared with CisPt group (*n*=8 each group).

Water intake and diuresis were increased in CisPt treated rats and remained increased in CisPt + 5-AIQ group 14 days after CisPt injection. Nevertheless, water balance was similar to control and CisPt groups and treatment with 5-AIQ induced a significant increase in this variable.

### Renal function studies

**Figure 1:**
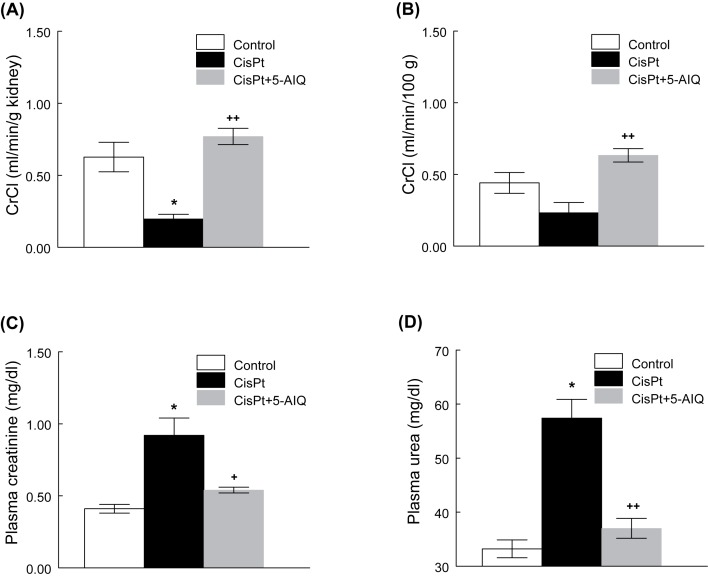
Creatinine clearance, plasma creatinine and urea concentrations in control, CisPt and CisPt + 5-AIQ groups 14 days after CisPt injection CrCl expressed as ml/min/g of kidney (**A**) and ml/min/100 g of rat (**B**) in control, CisPt, and CisPt + 5-AIQ groups 14 days after CisPt injection. Plasma creatinine (**C**) and urea (**D**) concentrations at the end of the experiment in control, CisPt, and CisPt + 5-AIQ groups. *P<0.001 compared with control group. ^+^*P*<0.01, ^++^*P*<0.001 compared with CisPt group (*n*=8 each group).

Figure 1 shows that CisPt treatment decreased CrCl 14 days after CisPt injection. Inhibition of PARP1 with 5-AIQ evoked an increase in CrCl in CisPt-treated rats and no significant differences were found between CisPt + 5-AIQ and control group. In plasma, CisPt group displayed higher concentrations of urea and creatinine that were also reduced at the level of control group with 5-AIQ treatment.

Regarding the urinary variables, CisPt augmented NAG excretion and glucosuria (Table 2). 5-AIQ treatment significantly decreased NAG excretion and glucosuria in comparison with CisPt-treated group. Proteinuria was similar in all experimental groups (Table 2).

**Table 2:** Urinary variables determined in Control, CisPt, and CisPt + 5-AIQ groups 14 days after CisPt injection

	Control	CisPt	CisPt + 5-AIQ
Proteinuria (mg/mg creatinine)	1.81 ± 0.22	2.05 ± 0.21	1.54 ± 0.16
Glucosuria (mg/mg creatinine)	0.37 ± 0.06	1.39 ± 0.48*	0.24 ± 0.03^‡^
NAG (mU/mg creatinine)	34.6 ± 5.01	90.5 ± 10.1^†^	37.2 ± 1.98^§^

Data are expressed as means ± S.E.M. *P<0.05. ^†^*P*<0.001 compared with control group. ^‡^*P*<0.01. ^§^*P*<0.001 compared with CisPt group (*n*=8 each group).

### Sodium handling

Treatment with CisPt increased plasma sodium concentration, and 5-AIQ reduced this variable to the level of control group (Table 3) 14 days after CisPt injection.

**Table 3:** Sodium handling in Control, CisPt, and CisPt + 5-AIQ groups 14 days after CisPt injection

	Control	CisPt	CisPt + 5-AIQ
Plasma Na (mEq/l)	139.7 ± 0.38	142.5 ± 0.55^†^	140.6 ± 0.45^‡^
NaTL (µEq/min/100 g)	61.5 ± 10.2	33.2 ± 10.3^†^	89.0 ± 6.63^§^
Natriuresis (µEq/100 g/day)	717 ± 75.9	477 ± 20.5*	453 ± 65*
RNa (µEq/min/100 g)	61.1 ± 10.1	32.9 ± 10.3^†^	88.7 ± 6.59^§^
NaFE (%)	0.86 ± 0.09	1.84 ± 0.50*	0.35 ± 0.03*^,‡^

Data are expressed as means ± S.E.M. Abbreviations: NaTL, sodium tubular load; RNa, reabsorpted sodium. *P<0.05. ^†^*P*<0.001 compared with control group. ^‡^*P*<0.01. ^§^*P*<0.001 compared with CisPt group (*n*=8 each group).

Despite the increased plasma sodium, sodium tubular load (NaTL), parameter that quantifies the amount of filtered sodium, was decreased in CisPt treated group due to the reduction in CrCl of these animals. Natriuresis was also lower than the control group as well as reabsorpted sodium (RNa), and NaFE was increased, indicating that CisPt-treated animals excreted a higher amount of sodium than should be expected when it is relativized to urine/plasma creatinine ratio due to a decrease in reabsorption process. However, CisPt + 5-AIQ group displayed an increased NaTL, which was similar to NaTL of control group. Despite the high value of NaTL, natriuresis and NaFE were decreased in CisPt + 5-AIQ group in comparison with control group. NaFE was also decreased when compared with CisPt group. Therefore, CisPt + 5-AIQ group displayed increased sodium reabsorption in comparison with CisPt treated group (Table 3).

**Figure 2:**
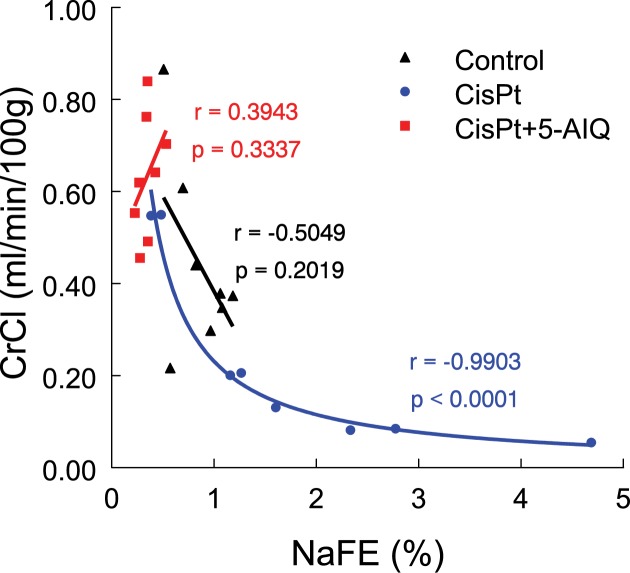
Correlation between NaFE and CrCl in Control, CisPt, and CisPt + 5-AIQ groups (*n*=8 each group) 14 days after CisPt injection

Strong negative correlation (*P*<0.0001, *r* = −0.9903) was found between NaFE and CrCl in CisPt treated group (Figure 2), but no correlation was found in Control (*P*=0.2019, *r* = −0.5049) or Cispt + 5-AIQ (*P*=0.3337, *r* = 0.3943) groups.

### Inhibition of PARP1

**Figure 3:**
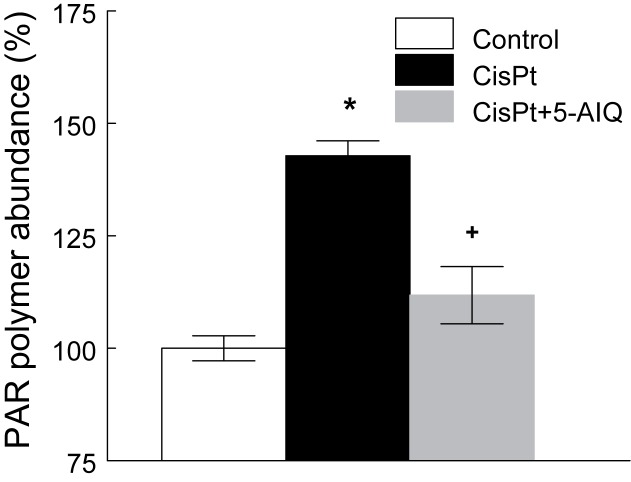
PAR polymer abundance in renal tissue in control, CisPt, and CisPt + 5-AIQ groups 14 days after CisPt injection Results were expressed as percentage of the mean absorbance of the control group. *P<0.001 compared with control group. ^+^*P*<0.001 compared with CisPt group (*n*=8 each group).

Fourteen days after CisPt injection, CisPt-treated rats displayed a significantly higher content of PAR polymer in renal tissue. Treatment with 5-AIQ completely prevented PAR polymer accumulation (Figure 3). These results indicate that CisPt treatment enhanced PARP-1 activity and demonstrate that 5-AIQ is a potent inhibitor of the enzyme.

### Renal fibrosis

**Figure 4:**
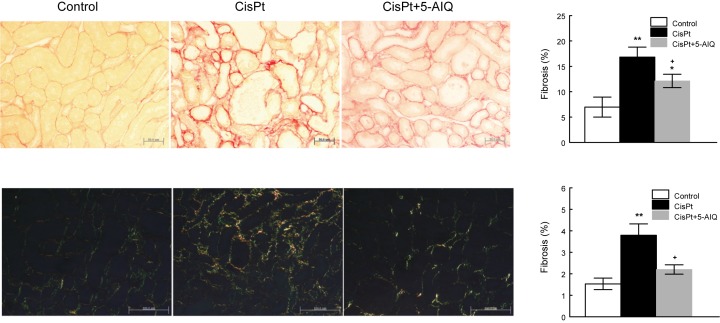
Representative photographs of Picro Sirius Red staining of kidneys from control, CisPt, and CisPt + 5-AIQ-treated rats 14 days after CisPt injection observed with standard light microscopy (top) and the same staining observed with polarized light (down) Bar 50 μm (top). Bar 100 μm (down). Renal fibrosis (%) quantitated with ImageJ software (right). *P<0.05, **P<0.001 compared with control group. ^+^*P*<0.05 compared with CisPt group (*n*=8 each group).

CisPt group displayed a higher percentage of interstitial fibrosis assessed by Sirius Red stain (Figure 4) 14 days after CisPt injection. 5-AIQ treatment significantly reduced interstitial fibrosis in comparison with CisPt-treated group (Figure 4).

### Renal expression of collagen-IV, α-SMA, and TGF-β1

**Figure 5:**
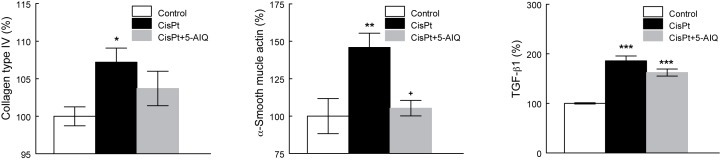
Collagen type IV, α-SMA, and TGF-β1 expression in renal tissue in control, CisPt, and CisPt + 5-AIQ groups 14 days after CisPt injection. Results were expressed as percentage of the mean absorbance of the control group. *P<0.05, **P<0.01, ***P<0.001 compared with control group. ^+^*P*<0.05 compared with CisPt group (*n*=8 each group).

**Figure 6:**
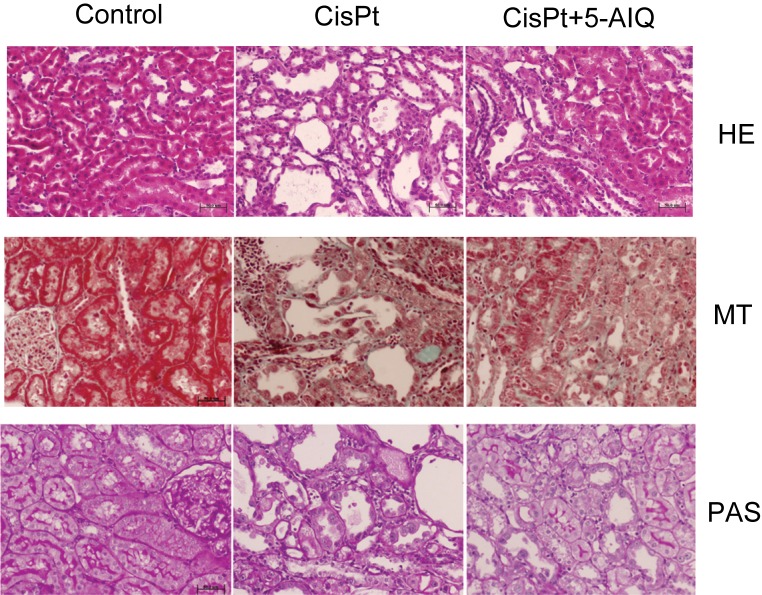
Hematoxylin and eosin (HE, top), Masson’s trichrome (MT, middle), and PAS stains in Control, CisPt, and CisPt + 5-AIQ-treated rats 14 days after CisPt injection Bar 50 μm. Original magnification 20×.

CisPt group showed a higher expression of some biochemical variables related to fibrosis: collagen-IV, α-SMA, and TGF-β1 (Figure 5). 5-AIQ treatment completely prevented α-SMA increase (Figure 6). Collagen-IV and TGF-β1 expressions in CisPt + 5-AIQ group were slightly lower than CisPt group but they did not reach statistical significance (Figure 5). It is remarkable that the expression of collagen-IV in CisPt + 5-AIQ group did not show statistical differences with control group indicating that 5-AIQ attenuated collagen expression to an intermediate level between both control and CisPt groups.

### Histopathological analysis

Renal lesions in control group were absent. No glomerular, tubulointerstitial, or vascular lesions were present in renal parenchyma. Histopathological examination of renal slices from CisPt-treated rats showed different alterations including relevant nuclear dysplasia and incipient acute tubular necrosis (Table 4 and Figure 6). The sections of the kidney from CisPt-treated rats exhibited marked dilation of proximal convoluted tubules in the corticomedullary junction with slogging of almost entire epithelium due to desquamation of tubular epithelium that induced a total absence of microvilli and loss of brush border in CisPt group. Also, mild tubular atrophy and apoptotic cells in tubular lumen were present in CisPt-treated group. CisPt group showed increased interstitial fibrosis in Picro Sirius Red and Masson’s trichrome staining. 5-AIQ significantly attenuated morphologic alterations induced by CisPt, indicating that PARP1 activity plays a role in the development of tubular lesions.

**Table 4:** Histopathological analysis in Control, CisPt, and CisPt + 5-AIQ groups 14 days after CisPt injection

	Control	CisPt	CisPt + 5-AIQ
S3 dysplasia	0	3 (2.5−3)^‡^	1 (1−2)^‡,║^
Acute tubular necrosis	0	2 (2−2)^‡^	1 (1−1.5)^†,║^
Apoptosis	0	2 (1.5−2.5)^‡^	1 (1−1)^‡,║^
Casts	0	1.5 (1−2)^†^	0^++^
Tubular atrophy	0	0	0
Tubular dilation	0	3 (2.5−3)^‡^	1 (1−2)^†,║^
Vacuolization	0	0	0
Inflammatory infiltrate	0	0 (0−1)	0
Tubular mitosis	0	1 (1−1.5)^‡^	1 (0−1)*^,§^

Data are expressed as median and interquartilic range. *P<0.05. ^†^*P*<0.01. ^‡^*P*<0.001 compared with control group. ^§^*P*<0.05. ^║^*P*<0.01 compared with CisPt group (*n*=8 each group).

## Discussion

The main findings of this work are that inhibition of PARP1 with 5-AIQ ameliorates renal dysfunction and prevents body weight loss, decreasing renal fibrosis and tubular lesions in CisPt treated rats 2 weeks after the treatment. These data for the first time demonstrate that improvements in renal function are maintained during recovery phase of CisPt-induced acute kidney injury (AKI). Thus, we found that 5-AIQ treatment significantly decreased urinary excretion of NAG, a marker of tubular damage [20] and glucosuria observed in the CisPt group. These last data agree in part with previous reports showing that pharmacological inhibition of PARP1 with PJ34, administered for a shorter period, 3 [16] or 5 [7] days after administration of CisPt protected against tubular necrosis, renal dysfunction, inflammatory response, and oxidative stress. Despite the positive results reported above, we also observed that tubular alterations and renal fibrosis were not completely prevented by 5-AIQ.

In consonance with our results, Kim and Padanilam [21] found that PARP1 deficiency in a model of *Parp1*-KO mice attenuated renal fibrosis and inflammation during unilateral ureteral obstruction but TGF-β1/Smad3 signaling pathway remained unchanged in *Parp1-*KO and WT mice. In our work, expression of TGF-β1 was also similar in CisPt and CisPt + 5-AIQ groups and this could explain that inhibition of PARP1 did not achieve a complete protection over renal fibrosis and tubular alterations in CisPt nephrotoxicity. In fact, TGF-β1 was demonstrated to play a role in initiating fibrogenesis in obstructive nephropathy [22] and it has been proposed as the key regulator of renal fibrosis [18].

CisPt is freely filtered at the glomerulus and taken up into renal tubular cells mainly by a transport-mediated process where it is partially metabolized into toxic species [23]. Hence, CisPt may develop multiple intracellular effects causing direct cytotoxicity and inducing apoptosis, which are not prevented by PARP1 inhibitors in mice [7]. This factor can also contribute to the incomplete protection that we have found in our study.

α-SMA expression was increased in CisPt group but it was intriguingly reduced to the level of control group in CisPt-AIQ treated rats, although these animals displayed renal fibrosis and high expression of TGF-β1. In this way, it has been recently published that only a minority of collagen-producing cells co-express α-SMA in the fibrotic kidney but they were equally capable of activating TGF-β1 suggesting that α-SMA is an inconsistent marker of contractile and collagen-producing fibroblasts in murine experimental models of organ fibrosis [24].

Multiple low doses of CisPt administered during 4 weeks also induced interstitial fibrosis and increased TGF-β1 and α-SMA expression in mice [25]. In this chronic model, kidney injury and interstitial fibrosis were attenuated by using PXS-4728A, an inhibitor of semicarbazide-sensitive amine oxidase. This antioxidant treatment decreased renal expression of both TGF-β1 and α-SMA in parallel. Besides, these authors observed that blood urea nitrogen and creatinine were decreased in these animals at the end of the study. These results demonstrate that oxidative stress plays a relevant role in CisPt-induced nephrotoxicity. Inhibition of PARP1 has been demonstrated to reduce oxidative stress in acute experiments [7,16]. Therefore, the reduction in oxidative stress can also contribute to the improvement in renal function that we have obtained in our study.

Improvement in renal function might be related with sodium handling in these animals. Other authors have described impaired sodium and water transport in CisPt-treated rats due to decreased mitochondrial function, decreased ATPase activity and altered cell cation content that alters solute transport [26,27]. Expression of aquaporins and several sodium transporters have been found to be decreased in this model [26,28]. This impaired sodium reabsorption has been related with increased vascular resistance and decreased glomerular filtration rate (GFR) secondary to tubular-glomerular feedback from increased NaCl delivery to the macula densa [27]. According to these findings in our work we have found a strong negative correlation between NaFE and CrCl in CisPt-treated animals indicating that a higher NaFE is accompanied by a lower GFR in these animals. On the contrary, we have found that 5-AIQ evoked a great increase in sodium reabsorption, probably due to the availability of ATP in renal tubular cells because of the inhibition of PARP1 activity. Increase in sodium reabsorption evoked by 5-AIQ would contribute to normalization of GFR of this group through several mechanisms: for one side, decreased sodium delivery at distal areas of the nephron would inhibit tubular-glomerular feedback increasing GFR and on the other side, increased sodium reabsorption could determine higher water reabsorption and intake as a homeostatic mechanism aimed to maintain plasma osmolality, increasing filtration fraction, and water balance in CisPt + 5-AIQ group. The participation of both mechanisms does not exclude the participation of other factors that could increase GFR in CisPt + 5-AIQ group. Our data show that correlation between NaFE and GFR is very slight or absent from control and CisPt + 5-AIQ groups indicating that other factors than NaFE contribute to increase GFR in these animals.

Another relevant aspect of this work is that 5-AIQ might be a therapeutic tool in patients that receives CisPt. 5-AIQ has been described as a potent inhibitor of poly(ADP-ribosyl)ation in mice [29]. Although quinolines are generally known as mutagenic and carcinogenic, 5-AIQ does not possess genotoxic activity both *in vitro* and *in vivo* systems [30]. Our results demonstrate that 5-AIQ might have a potential therapeutic value to prevent CisPt nephrotoxicity and the development of fibrosis that has been found to be associated with CisPt treatment [31]. In fact, the second phase of CisPt nephrotoxicity, which is characterized by decreased GFR and impaired NaCl transport does not respond to any drug [32,33], being therefore of clinical interest to evaluate the effects of this substance in other animal models and in patients. Furthermore, these studies should investigate if treatment with 5-AIQ might affect the antitumoral effect of CisPt.

In summary, this work demonstrates that PARP1 activity participates in most of the nephrotoxic effects evoked by CisPt. The administration of 5-AIQ, a potent PARP1 inhibitor improves renal function and fibrosis during recovery phase of CisPt-induced AKI in rats. These findings can be of clinical interest to prevent renal dysfunction in CisPt-treated patients.

## Abbreviations

AKI: acute kidney injury
CisPt: cisplatin
CrCl: creatinine clearance
GFR: glomerular filtration rate
NaFE: sodium fractional excretion
NAG: N-acetyl-β-d-glucosaminidase
NaTL: sodium tubular load
PARP1: poly(ADP-ribose) polymerase-1
TGF-β1: transforming growth factor-β1
α-SMA: α-smooth muscle actin
5-AIQ: 5-aminoisoquinoline
